# Importance of Continuous Sequential Chemotherapy and Multimodal Treatment for Advanced Testicular Cancer: A High-Volume Japanese Center Experience

**DOI:** 10.1097/MD.0000000000000653

**Published:** 2015-03-20

**Authors:** Terukazu Nakamura, Takashi Ueda, Masakatsu Oishi, Hiroyuki Nakanishi, Takumi Shiraishi, Atsuko Fujihara, Yasuyuki Naito, Kazumi Kamoi, Yoshio Naya, Fumiya Hongo, Koji Okihara, Tsuneharu Miki

**Affiliations:** From the Department of Urology, Graduate School of Medical Science, Kyoto Prefectural University of Medicine, Kyoto, Japan.

## Abstract

Patients with “difficult-to-treat” advanced testicular cancer can require multiple therapies. We retrospectively assessed our patients with advanced germ cell tumors (GCTs) and characterized the clinical efficacy, outcomes, and factors affecting overall survival (OS).

Two hundred fifty-three patients with advanced GCTs were treated at Kyoto Prefectural University of Medicine, Kyoto, Japan, from June 1998 to September 2013. Of 253 patients, 142 patients had salvage chemotherapy.

As first-line therapy, bleomycin, etoposide, and cisplatin, and etoposide and cisplatin therapies were performed in 234 cases (92.5%). As second-line therapy, etoposide, ifosfamide, and cisplatin/vinblastine, ifosfamide, and cisplatin, and paclitaxel, ifosfamide, and cisplatin/paclitaxel, ifosfamide, and nedaplatin therapies were carried out in 44 and 59 cases, respectively. Furthermore, 111, 72, 44, and 28 cases had third, fourth, fifth, and sixth-or-later-line chemotherapy, respectively. Five-year OS rate stratified by chemotherapy line was 95.5% in the first line, 89.4% in the second line, 82.1% in the third line, 45.1% in the fourth line, and 58.9% in the fifth or after line. A statistical significant difference was found when comparing fourth-or-after-line versus first to third-line therapy. Additional procedures were performed, including retroperitoneal lymph node dissection (RPLND) (n = 168), extra-RPLN resection (n = 114), and external beam radiotherapy/stereotactic radiotherapy (n = 78).

Multivariate analysis showed that factors predicting better outcomes were in serum tumor marker (STM) normalization, RPLND, and extra-RPLN resection.

Good outcomes were obtained in patients who completed chemotherapy up to third line. After fourth-line chemotherapy, approximately 50% of “difficult-to-treat” patients could be cured with normalization of STM levels and residual mass resection. Continuous or sequential chemotherapy with multimodality therapy is important for patients with “difficult-to-treat” advanced GCTs. Effective chemotherapy after third line should be developed.

## INTRODUCTION

Advanced metastatic germ cell tumors (GCTs) can be cured in 80% of cases.^1^ Survival improvement in poor prognosis should be noted in the modern chemotherapy era. Population-based analysis by Swedish–Norwegian group showed overall survival (OS) was 67.4% in poor prognosis.^2^ However, 20% to 30% of cases of advanced GCTs require salvage chemotherapy, such as vinblastine, ifosfamide, and cisplatin (VeIP), etoposide, ifosfamide, and cisplatin (VIP), high-dose chemotherapy (HDCT), and paclitaxel, ifosfamide, and cisplatin (TIP).

Although VIP and VeIP therapy have been used as standard salvage therapy for 2 decades, no evidence of disease (NED) rate was approximately 30%.^3,4^ These results led investigators to develop other therapies. HDCT was made possible by the development of peripheral stem cell blood harvest and transfusion. Two randomized control trials (RCTs) of HDCT as first salvage therapy (second-line) failed to show that HDCT was superior to standard-dose salvage therapy.^5,6^ In addition, after failing HDCT therapy, it is very difficult to continue treatment.^7^

TIP therapy as first salvage chemotherapy, introduced by Motzer in 2000,^8^ results in a very good response rate (RR) in cases with specific prognostic features (eg, testis primary and first relapse after previous complete response [CR]). Other investigators tested TIP as second-line therapy for patients with bleomycin, etoposide, and cisplatin (BEP) failure and reported relatively good results with manageable adverse events.^9^ Therefore, TIP has been recognized as a standard first salvage chemotherapy, especially for the patients with favorable prognostic features.

Other salvage chemotherapies have been developed for advanced GCTs. Some of them seem to be good candidates for second-line treatment or for use after salvage chemotherapy. Gemcitabine and irinotecan in combination with cisplatin or its derivatives resulted in relatively good cure rates.^10,11^ A regimen including gemcitabine or irinotecan has mainly been used as third-line therapy or after salvage chemotherapy.

Guidelines equally recommend VeIP/VIP, TIP, and HDCT as second-line therapy.^12–14^ With regard to third-line therapy, gemcitabine-containing regimens might be good candidates, but no specific regimen was mentioned recommended by the guidelines.

To our knowledge, there are no definitive data to guide the sequential treatment regimens for patients with “difficult-to-treat” advanced testicular cancer. In addition, there are no reports that studied the entire treatment sequence and survival from induction chemotherapy to completion of treatment. Thus, the goal of this retrospective study was to assess the response and OS of sequential chemotherapy and additional modalities for patients with advanced GCT at each step of treatment, regardless of the chemotherapy regimen.

## PATIENTS AND METHODS

We retrospectively assessed 253 patients having GCT with metastasis treated in our institution from June 1998 to September 2013. Patient characteristics are summarized in Table 1. Of 253 patients, 111 patients had only first-line chemotherapy (induction group) and 142 patients had second-line-or-later chemotherapy (salvage group). The salvage group had worse initial clinical stage and International Germ Cell Cancer Collaborative Group risk criteria compared with induction group. This study was approved by the Institutional Review Board of Kyoto Prefectural University of Medicine, Kyoto, Japan, and written informed consent for each treatment was obtained from all patients.

**TABLE 1:**
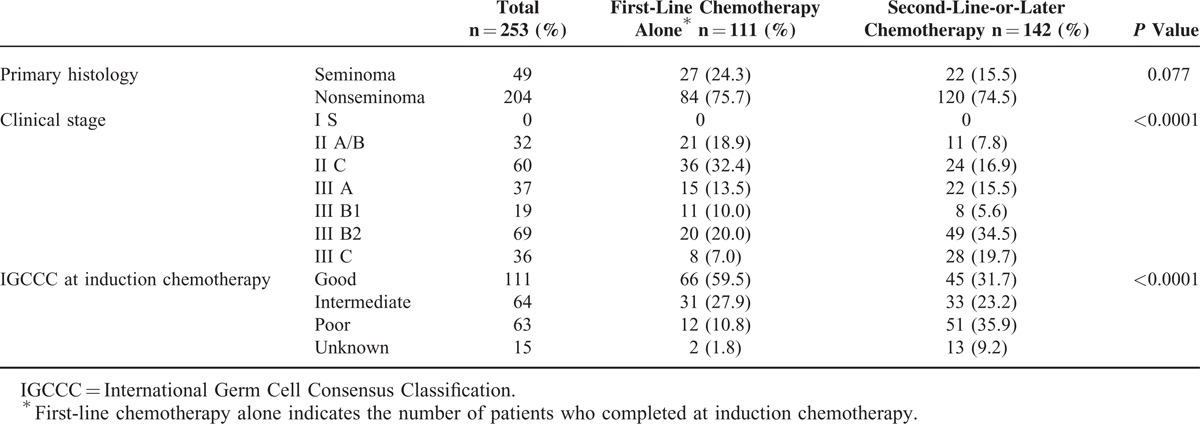
Patient Characteristics

### Treatment Strategy

We treated advanced patients with GCT according to our treatment strategy, as follows:

1. Patients who achieved tumor marker normalization with residual mass had residual mass resection such as retroperitoneal lymph node dissection (RPLND), cervical lymph node dissection, and visceral metastatic site resection.
2. Patients without serum tumor markers (STMs) normalization after chemotherapy were treated with a subsequent chemotherapy. Our salvage chemotherapy strategy was as follows: second-line (TIP or paclitaxel, ifosfamide, and nedaplatin [TIN], third-line (irinotecan and cisplatin [IrP] or irinotecan and nedaplatin [IrN], fourth-line (paclitaxel, gemcitabine, and cisplatin [TGP] or paclitaxel, gemcitabine, and nedaplatin [TGN]), and fifth-line-or-after (clinical trial). For the patients who had already been treated at another institution, our first selection was a therapy that had not been used for that patient before.
3. If tumor marker level had been progressively decreasing, the current chemotherapy was continued. On the contrary, when tumor marker levels increased or if progressive disease (PD) was seen by radiographic studies, the patient was switched to a different chemotherapy.
4. Extrabeam radiotherapy and/or radiofrequency ablation as alternative focal therapy were performed, even for nonseminoma patients, in the curative or palliative setting according to the physician's decision.
5. For brain metastasis, stereotactic radiotherapy, such as γ-knife or cyber-knife, was selected first. When dissemination or relapse occurred, whole brain irradiation was considered.
6. When viable cancer, not including teratoma, was found at residual mass resection, an additional 2 cycles of chemotherapy was typically performed.

### Response Assessment

Response assessment was carried out following every other cycle according to the Response Evaluation Criteria in Solid Tumors Criteria ver1.1. CR was defined as the disappearance of all clinically detectable disease and as STM normalization. Partial response (PR) with STM normalization (PRm−) was defined as ≥30% reduction of the tumor size and normalization of previously elevated tumor markers. PR without STM normalization was considered marker-positive PR (PRm+). No change of visible disease (NC) was also classified as NCm− (NC with STM normalization) and NCm+ (NC without STM normalization). If significant STM elevation (>50%) and/or radiological progression (>25%) occurred after the beginning of treatment, the patient was classified as having PD.

RR was defined as CR + PR regardless of STM normalization. STM normalization (STMn) was defined as normal STM regardless of tumor shrinkage.

### Statistical Analysis

Survival analysis was performed using the Kaplan–Meier method, and statistical difference was tested by the log-rank test. The primary endpoint was OS. Univariate and multivariate analysis were performed using JMP 10 software (SAS Institute, Inc, Cary, NC). Survival was confirmed by last visit to our institution or telephone.

## RESULTS

### Sequential Chemotherapy

Table 2 shows the treatment sequence at our department with other additional procedures. Briefly, BEP or etoposide and cisplatin (EP) therapy was chosen in 234 patients as induction chemotherapy. As second-line therapy, VIP or VeIP were selected in 44 patients, and TIP or TIN was done in 59 patients. With regard to third-line therapy, the most commonly selected therapy was TIP or TIN therapy in 53 patients, and IrP or IrN were the second most commonly selected therapy (18 patients). The most common trend of sequential chemotherapy for “difficult-to-treat” GCTs was BEP/EP-TIP/TIN-IrP/IrN-TGP/TGN. The second most common sequential chemotherapy was BEP/EP-VeIP/VIP-TIP/TIN-IrP/IrN.

**TABLE 2:**
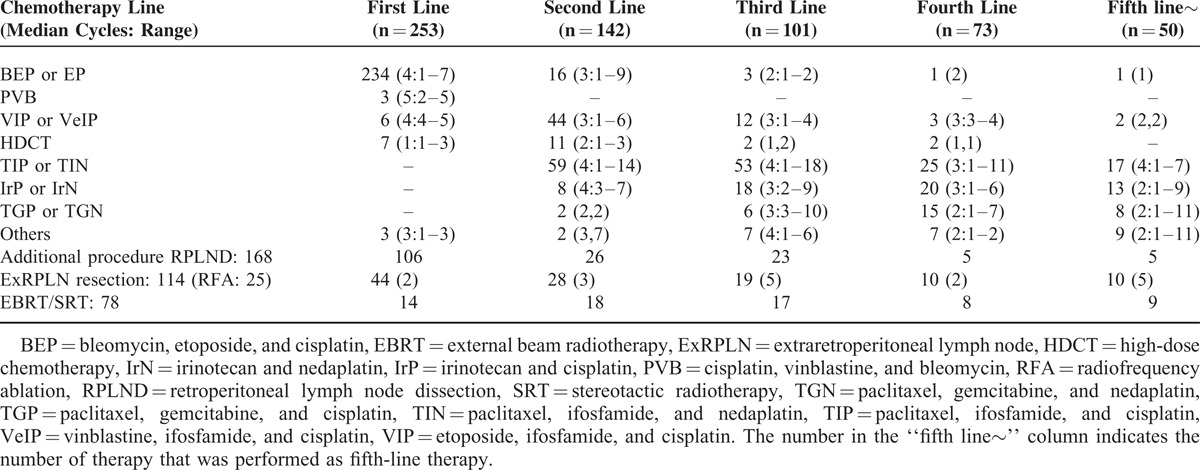
Chemotherapy Sequence in Our Department, n = 253

With regard to additional procedures during or after chemotherapy, 150 surgeries and 14 radiation therapies were performed during or after first-line therapy. Out of 169 RPLND carried out, 156 were integrated with first, second, and third-line therapy.

### Response Rate

The RRs (CR + PR) for the second, third, and fourth-line therapies are shown in Table 3. RR and STMn were obtained in 69.5% and 61.1%, respectively, in response to TIP/TIN therapy. VIP or VeIP also showed good STMn rate (50.0%). In third-line therapy, STMn occurred in 52.8% and 41.7% in response to TIP/TIN and VIP/VeIP, respectively. Even in the fourth-line setting, TIP/TIN resulted in a good STMn rate (44.0%).

**TABLE 3:**
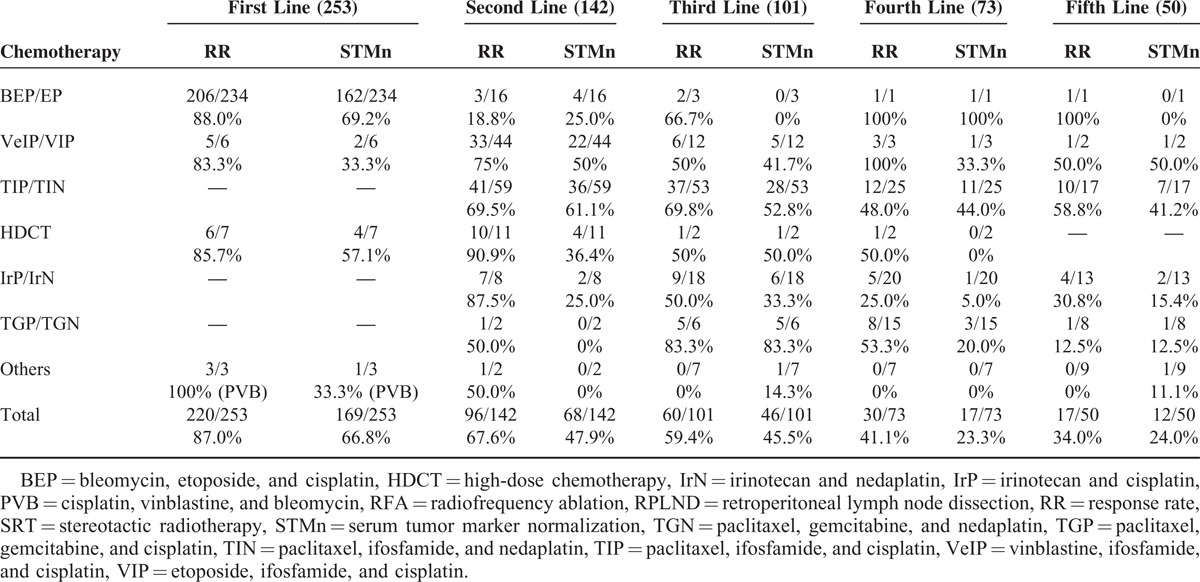
RR and STM Normalization Rate at Each Line Stratified by Regimen

### Overall Survival

**FIGURE 1:**
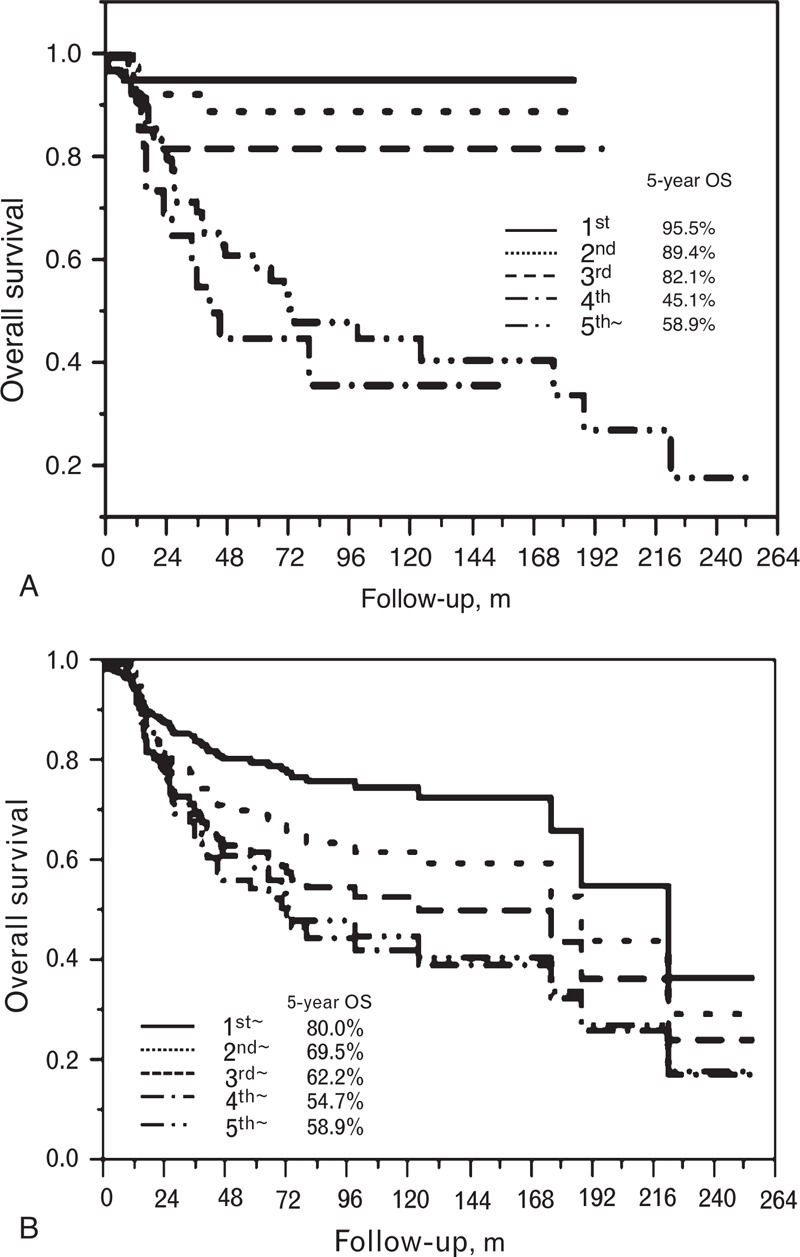
(A) OS, stratified by required chemotherapy. Kaplan–Meier curve by required chemotherapy line. For example, 111 patients finished first-line chemotherapy only and showed 5-year OS of 95.5%. A statistical difference was found when comparing the following pairs of groups: third-line and first-line (hazard ratio [HR], 3.6; 95% confidence interval [CI], 1.0–12.9; *P* = 0.049), fourth-line and first-line (HR, 14.8; 95% CI, 5.6–46.1; *P* < 0.0001), fifth-line and first-line (HR, 11.5; 95% CI, 4.8–33.8; *P* < 0.0001), fourth-line and second-line (HR, 7.1; 95% CI, 2.5–25.3; *P* = 0.0001), fifth-line and second-line (HR, 5.5; 95% CI, 2.2–18.7; *P* = 0.0001), fourth-line and third-line (HR, 4.1; 95% CI, 1.5–12.8; *P* = 0.004), and fifth-line and third-line (HR, 3.2; 95% CI, 1.3–9.4; *P* = 0.007). (B) OS, stratified by chemotherapy line. Kaplan–Meier curve by chemotherapy line. For example, 101 patients had third-line-or-later chemotherapy and showed 5-year OS of 62.2%. A statistical difference was found when comparing the following pairs of groups: second-line and first-line (HR, 1.6; 95% CI, 1.1–2.3; *P* = 0.018), third-line and first-line (HR, 2.0; 95% CI, 1.4–3.0; *P* = 0.0004), fourth-line and first-line (HR, 2.6; 95% CI, 1.7–3.8; *P* < 0.0001), fourth-line and second-line (HR, 1.6; 95% CI, 1.1–2.4; *P* = 0.022), and fifth-line and first-line (HR, 2.4; 95% CI, 1.5–3.7; *P* = 0.0004). OS = overall survival.

OS stratified by required chemotherapy line is shown in Figure 1A. In this analysis, second-line meant that the final chemotherapy was second-line therapy. Thus, the number of patients who completed therapy as first line, second line, third line, fourth line, fifth line, and sixth line or after was 111, 41, 28, 23, 18, and 32 cases, respectively. Figure 1B shows OS stratified by chemotherapy line. In this analysis, “third-line∼” represented patients who had third-line-or-after chemotherapy (eg, fourth, fifth, and sixth-line-or-after therapy). In the patients with RPLND, residual viable cancer was found in 20.5% and teratoma was found in 23.7%. The 5 and 10-year OS rate was 91.5% and 87.9%, respectively.

In the search for the best sequence from second line to third line, we could not identify a sequence that was statistical significant superior to others in any combination. The sequence of second line and third line chemotherapy with VeIP/VIP and TIP/TIN had no significant difference, compared with other combinations, such as TIP/TIN and IrP/IrN (hazard ratio; 1.02, 95% confidence interval; 0.39–3.20, *P* = 0.97).

### Statistical Analysis

Univariate and multivariate analysis are shown in Tables 4 and 5, respectively. Univariate analysis showed significant differences in each category of the International Germ Cell Consensus Classification, each clinical stage, doing RPLND or not, doing extra-RPLN resection or not, with or without radiation, and achieving STM normalization or not. Multivariate analysis showed significant differences in each clinical stage, doing RPLND or not, doing extra-RPLN resection or not, with or without radiation and achieving STM normalization or not.

**TABLE 4:**
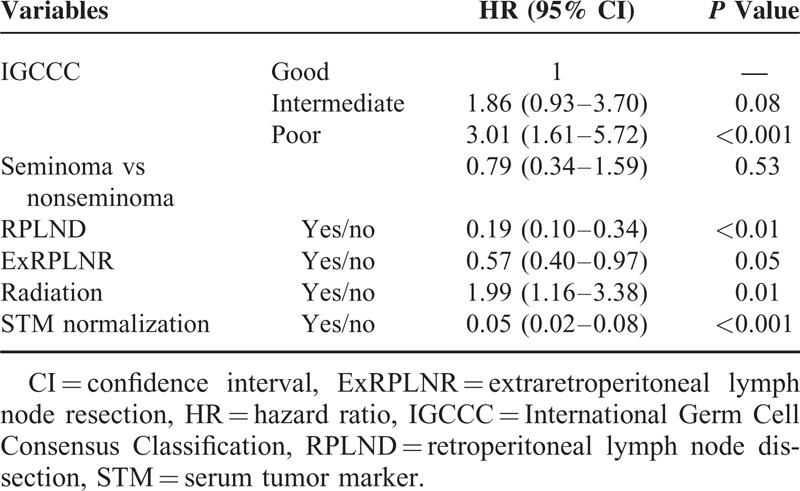
Univariate Analysis of Factors Related to Overall Survival

**TABLE 5:**
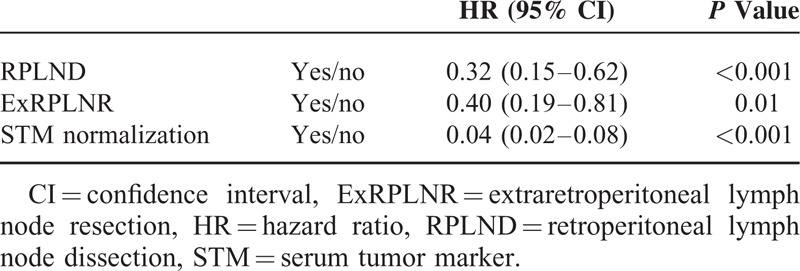
Multivariate Analysis of Factors Related to Overall Survival

## DISCUSSION

BEP therapy is widely used as first-line therapy for advanced testicular cancer. On the contrary, only 2 phase III trials of HDCT in the second-line setting have been performed,^4,5^ and there are no RCTs of conventional-dose salvage chemotherapy. Therefore, guidelines do not recommend salvage chemotherapy.^12–14^

Our institutional strategy for advanced GCT was BEP/EP as induction chemotherapy, TIP/TIN (substitute nedaplatin for cisplatin) as second-line, IrP/IrN as third-line, and TGP/TGN as fourth-line therapy. Therefore, TIP/TIN was the most common regimen selected as second line. The second most common choice for second-line therapy was VIP/VeIP. This is compatible with guidelines’ recommendation. In the third-line or later setting, paclitaxel, irinotecan, or gemcitabine were selected (Table 3).

As shown in Table 6, after introducing ifosfamide, VIP or VeIP has been used as second-line therapy. Although its efficacy was relatively good, long NED could be achieved in about 30%, which is suboptimal.^3,4^ This led investigators to introduce HDCT with stem cell transfusion. Two RCTs did not show superiority of HDCT to conventional dose chemotherapy (CDCT).^5,6^ These unsatisfactory results led to the development of new regimens with paclitaxel,^8,9,15^ gemcitabine,^11,16–18^ and irinotecan.^10,19,20^ Most reports that have investigated chemotherapy regimens as second or third-line therapy showed relatively good results (OS of 20%–50%). Paclitaxel-containing therapy resulted in good survival rates when used in the second and third-line setting. From these results, the efficacy of HDCT was thought to be very limited, and paclitaxel-containing chemotherapy was promising as second or third-line therapy. In fact, our data showed that TIP/TIN resulted in a good response and in tumor marker normalization. For third-line therapy, our results showed an OS rate of 62.2%, which was better than the OS (20%) seen in previous studies.^11,15,16,19^ In addition, sequential chemotherapy, even after fourth line, produced a good RR and a good OS rate.

**TABLE 6:**
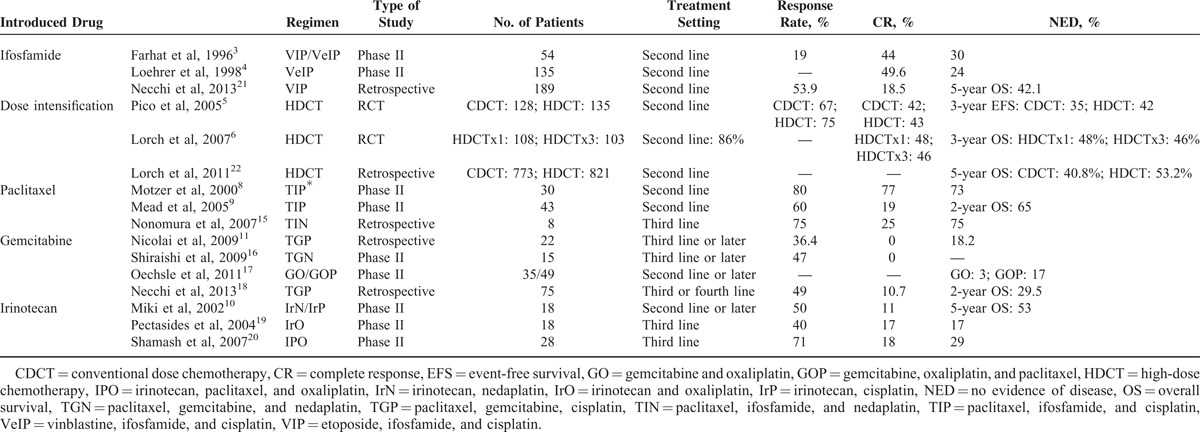
Reported Salvage Chemotherapy

Recently, Necchi et al^21^ used modified VIP as second-line therapy in a large cohort. This report showed that 2-year progression-free survival (PFS) and 5-year OS was 34.3% and 42.1%, respectively. Another large retrospective study by Lorch et al^22^ showed better second-line outcomes with HDCT than with CDCT. PFS in all subgroups was better after HDCT than CDCT and OS was better than CDCT in all except 1 (low risk).

HDCT seemed to be superior to CDCT. However, since CDCT and HDCT regimen were varied and selection bias must exist in a retrospective nature, definitive conclusion could not be obtained. Therefore, superiority of CDCT or HDCT should be reevaluated in a prospective RCT for patients with poor prognostic features, such as TIGER (Randomized Phase III Trial of Initial Salvage Chemotherapy for Patients with Germ Cell Tumors) study.^23^

Despite the absence of evidence from RCTs, TIP is effective first salvage chemotherapy. Furthermore, treatment options are more complicated in the third-line or later setting. Our sequential treatment strategy was comparable or better in terms of OS compared with reported literatures in each salvage chemotherapy line. These observations highlight the importance of sequential continuous chemotherapy integrated with residual mass resection. With regard to the best sequence up to third-line chemotherapy, the combination of TIP (microtubule polymerization inhibitor) as second line and gemcitabine (antimetabolite) as third line seems to be the most common sequence in the literature. Irinotecan (topoisomerase I inhibitor) was also a good candidate as second-line-or-later chemotherapy. However, we could not identify the best sequence in this series. Further investigation using anticancer agents with different mechanisms is required.

Residual mass resection is an important and essential part of GCT treatment.^12–14^ The rate of persistent viable cancer and teratoma are 10% and 40%, respectively, in the modern chemotherapy era. Disease-specific survival rate is reported to be 75% in the literature.^24–27^ Our data involving patients with complicated background showed that viable cancer was found in 20.5% and that teratoma was found in 23.7%. The 5 and 10-year OS rate was 91.5% and 87.9%, respectively. These results are comparable to those from the previous reports.^24–27^

The results of univariate and multivariate analysis suggest that it is very important to continue chemotherapy sequentially until STM normalization and resect residual mass, including RPLN and extra-RPLN, after finishing chemotherapy. Guidelines emphasize this point.^12–14^

This study has several limitations. First, this was a retrospective study of unselected patients. Second, since multiple centers contributed and only a small number of patients started induction chemotherapy at our institution, the treatment strategy was inconsistent, and patient backgrounds were heterogeneous in each treatment. Third, prognostic factors were unadjusted when comparing RRs. This is the first report of sequential continuous treatment that described the response and survival in each treatment line in the real clinical world. We believe these data show the importance of sequential treatment for advanced GCTs, despite the various study limitations.

In conclusion, this is the first report of multimodal and chemotherapeutic sequences for patients with advanced GCT. Sequential treatment for advanced GCTs, especially “difficult-to-treat” GCTs, is very important to improve outcomes. Very high cure rates were achieved in patients who completed chemotherapy up to third-line therapies. In addition, even in heavily treated patients who had fifth or sixth-line therapy, continuous treatment might save their lives if accompanied by additional modalities. More systematic trials that include third-line or fourth-line treatment are needed.
